# The values of applying classification and counts of white blood cells to the prognostic evaluation of resectable gastric cancers

**DOI:** 10.1186/s12876-018-0812-0

**Published:** 2018-06-28

**Authors:** Yin-Ling Wang, Xin-Xin Ge, Yi Wang, Meng-Dan Xu, Fei-Ran Gong, Min Tao, Wen-Jie Wang, Liu-Mei Shou, Kai Chen, Meng-Yao Wu, Wei Li

**Affiliations:** 1 Department of Oncology, the First Affiliated Hospital of Soochow University, Suzhou, 215006 China; 2 Department of Hematology, the First Affiliated Hospital of Soochow University, Suzhou, 215006 China; 3 PREMED Key Laboratory for Precision Medicine, Soochow University, Suzhou, 215021 China; 4 Department of Radio-Oncology, Nanjing Medical University Affiliated Suzhou Hospital, Suzhou, 215001 China; 5 Department of Oncology, the First Affiliated Hospital of Zhejiang Chinese Medicine University, Hangzhou, 310006 China; 6 Comprehensive Cancer Center, Suzhou Xiangcheng People’s Hospital, Suzhou, 215000 China

**Keywords:** Prognosis, Overall survival (OS), White blood cell (WBC), Gastric cancer (GC)

## Abstract

**Background:**

The classifications and counts of white blood cells (WBCs) have been proved to be able to be used as prognostic markers in cancer cases. The present study investigated the potential values of the classifications and counts of WBC, including lymphocyte (LY), monocyte (MO), neutrophil (NE), eosinophil (EO), and basophil (BA) in the prognosis of resectable gastric cancers (GCs).

**Methods:**

This retrospective study recruited 104 resectable GC cases which were pathologically confirmed. The patients were divided into two groups according to the median pre-treatment values. To evaluate the changes in WBC counts and classification after treatment, we introduced the concept of post/pre-treatment ratios (≤ 1 indicated count was not increased after therapy, while > 1 suggested increased count).

**Results:**

Pre-treatment NE and total WBC counts were negatively correlated with overall survival (OS). Surgery significantly decreased the level of NE count, but increased the level of EO, whereas had no effect on the levels of LY, MO, BAor total WBC. Adjuvant chemotherapy significantly decreased the level of BA. Whole course of treatment (surgery combined with adjuvant chemotherapy) had no significant effect on the counts of LY, MO, NE, EO, BA or total WBC. Post/pre-treatment ratios of LY, MO NE, EO, BA and total WBC levels had no effects on OS. Univariate analysis indicated that AJCC stage (III) and higher level of pre-treatment total WBC count were prognostic factors affecting OS. Multivariate Cox regression analysis revealed that AJCC stage (III) and higher level of pre-treatment total WBC count were independent prognostic factors.

**Conclusions:**

Pre-treatment NE count and pre-treatment total WBC count may be potential prognostic factors for the prognostic evaluation of GCs.

## Background

Gastric cancer (GC) is the fourth most common cancer in the world and the second essential factor to death of cancers [1, 2]. According to the cancer statistics in 2017, new gastric cancer cases numbered almost 28,000, and more than 10,960 deaths were caused by gastric cancer in united states [2]. Although both the lethality and morbidity of GC have decreased in recent years, the 5-year survival rate remains quite low still [3]. Therefore, it is important to identify reliable predictive factors for the prognosis of GC patients.

Total white blood cell (WBC) count which is often elevated during infections, is one of the nonspecific markers of inflammation and can be linked with some particular kinds of cancers [4]. WBC can be divided into five types, including lymphocyte (LY), monocyte (MO), neutrophil (NE), eosinophil (EO), basophil (BA) [5]. LY can respond to the host’s immune response, and low level of LY has been reported in various cancers [6, 7]. MO is produced in the bone marrow and stored in bone marrow, liver, as well as lymph nodes. MO plays critical role in systemic inflammatory response and steady-state immune-surveillance [8]. NE responds to infection and injury [9]. In addition, an elevated count of blood NE has been shown to predict poor survival in advanced GC [10] and esophageal cancer [11]. Besides, Sam C. Wang et al. proved that pretreatment NE to LY ratio independently predicts disease-specific survival in patients with resectable gastroesophageal junction and gastric adenocarcinoma [12]. EO is uncommon in healthy individuals, however, it is associated with allergies, helminth infections and some inflammatory states. Moreover, previous studies have shown a close association between EO level and the prognosis of GC patients [13]. BA, derived from bone marrow, is the rarest granulocytes and occupied less than 1% of peripheral leukocytes [14]. BA is well recognized to play a crucial role in protection against infections with parasites, allergy and autoimmunity [15, 16].

The present study investigated whether the classification and counts of WBCs can be served as prognostic indicators in patients with the resectable GC.

## Methods

### Subjects and inclusion criteria

This study was conducted as a retrospective investigation of resectable GC patients that had been referred to the First Affiliated Hospital of Soochow University (Jiangsu, China) between June 2007 and July 2016. Approval for the study was granted by the Medical Ethics Committees of the First Affiliated Hospital of Soochow University. Clinical and pathological records of all the patients participating in the study were reviewed periodically.

In total, 104 resectable GC patients were recruited in this study. All cases were confirmed by surgery and pathology. Patient characteristics are detailed in Table 1. The median age of the 104 patients was 60 years (range, 30–77 years). 77 patients were male and 27 were female. The staging of cancer was made according to tumor-nodulus-metastases (TNM) classification and classified through the American Joint Committee on Cancer (AJCC) recommendations. The prognostic analyses were performed regarding overall survival (OS).

**Table 1:** Clinicopathologic features

Clinicopathologic features	n	LY				MO				NE				EO				BA				total WBC			
		Low (n)	High (n)	χ2	*P* value	Low (n)	High (n)	χ2	*P* value	Low (n)	High (n)	χ2	*P* value	Low (n)	High (n)	χ2	*P* value	Low (n)	High (n)	χ2	*P* value	Low (n)	High (n)	χ2	*P* value
Gender	104																								
Male	77	37	40	0.45	0.502	35	42	3.599	0.058	32	45	8.454	0.004**	34	43	4.052	0.044*	39	38	7.836	0.005**	34	43	4.052	0.044*
Female	27	15	12			18	9			20	7			18	9			22	5			18	9		
Age (years)																									
≤ 60	54	25	29	0.616	0.432	23	31	3.148	0.076	26	28	0.154	0.695	29	25	0.616	0.432	37	17	4.507	0.034*	28	26	0.154	0.695
> 60	50	27	23			30	20			26	24			23	27			24	26			24	26		
Tumor size (cm)																									
≤ 5	79	44	35	4.265	0.039*	43	36	1.582	0.208	41	38	0.474	0.491	39	40	0.053	0.819	47	32	0.096	0.757	38	41	0.474	0.491
> 5	25	8	17			10	15			11	14			13	12			14	11			14	11		
Depth of invasion																									
T1, T2	5	1	4	0.84	0.359	2	3	0.002	0.965	5	0	–	0.028*	2	3	0	1	3	2	0.162	0.687	2	3	0	1
T3, T4	99	51	48			51	48			47	52			50	49			58	41			50	49		
Lymphonodus metastasis																									
N0, N1	28	17	11	1.759	0.185	16	12	0.586	0.444	19	9	4.887	0.027*	16	12	0.782	0.377	15	13	0.408	0.523	19	9	4.887	0.027*
N2	76	35	41			37	39			33	43			36	40			46	30			33	43		
AJCC stage																									
I,II	30	17	13	0.75	0.387	19	11	2.582	0.108	21	9	6.746	0.009**	15	15	0	1	15	13	0.624	0.43	19	11	2.998	0.083
III	74	35	39			34	40			31	43			37	37			46	28			33	41		

*P<0.05; **P<0.01

### Blood samples

Peripheral venous blood (5–7 ml) was collected into a sterile ethylenediaminetetraacetic acid (EDTA) tube. All blood samples were fasted and obtained between 6:30 and 7:30 a.m. in order to standardize the known impact of circulating hormones (circadian rhythm) on the number and subtype distribution of the various WBC indices. Hematological parameters were analyzed within 30 min after collection using a hematology analyser (Sysmex XE-2100; Sysmex, Kobe, Japan). LY, MO, EO, NE, BA and total WBC counts were recorded in Table 1. The patients were divided into two groups according to the median counts of LY, MO, EO, NE, BA or total WBC. The post/pre-treatment ratios were defined as the rate of pre-therapy blood parameters count and the corresponding ones obtained after therapy.

### Evaluation

Computed tomography (CT) scan was performed for the assessment of response every 2 months and evaluated according to the criteria of Response Evaluation Criteria in Solid Tumors (RECIST) 1.1.

### Follow-up

Survival time was measured from the date of chemotherapy until death or last clinical evaluation. The prognostic analyses were performed regarding OS. OS was defined as the time from the diagnosed date to death from any cause.

### Statistical analysis

All statistical analyses were performed using SPSS 19.0 software (Chicago, USA). For analysis of survival data, Kaplan-Meier curves were constructed, and statistical analysis was carried out using the log-rank test. The associations between blood parameters status and clinicopathologic features were explored by the χ2 tests. The relationships between changes in the blood parameters status and treatments were assessed by the t tests. Multivariate logistic regression model was employed to identify the independent risk factors associated with resectable GC. All values of *P* < 0.05 were considered statistically significant.

## Results

### The clinicopathologic features of resectable GC patients

Table 1 summarizes the clinicopathological characteristics and their relationship to the levels of LY, MO, NE, EO, BA and total WBC. The gender was significantly different in levels of NE(*P* = 0.004), EO(*P* = 0.044), BA(*P* = 0.005) and total WBC(*P* = 0.044) compared to those of LY and MO (both *P* >  0.05). The age was significantly different in levels of BA (*P* = 0.034) compared to those of LY, MO, NE, EO and total WBC (all *P* >  0.05). The tumor size was significantly different in levels of LY (*P* = 0.039) compared to those of MO, NE, EO,BA and total WBC (all *P* >  0.05). The depth of invasion was significantly different in levels of NE (*P* = 0.028) compared to those of LY, MO, EO,BA and total WBC (all *P* >  0.05). The Lymphonodus metastasis was significantly different in levels of NE and total WBC (both *P* = 0.027) compared to those of LY, MO, EO and BA (all *P* >  0.05). The AJCC stage was significantly different in levels of NE (*P* = 0.009) compared to those of LY, MO, EO,BA and total WBC (all *P* >  0.05).

### Pre-treatment NE and total WBC count were correlated with outcomes of resectable GC patients

**Fig. 1:**
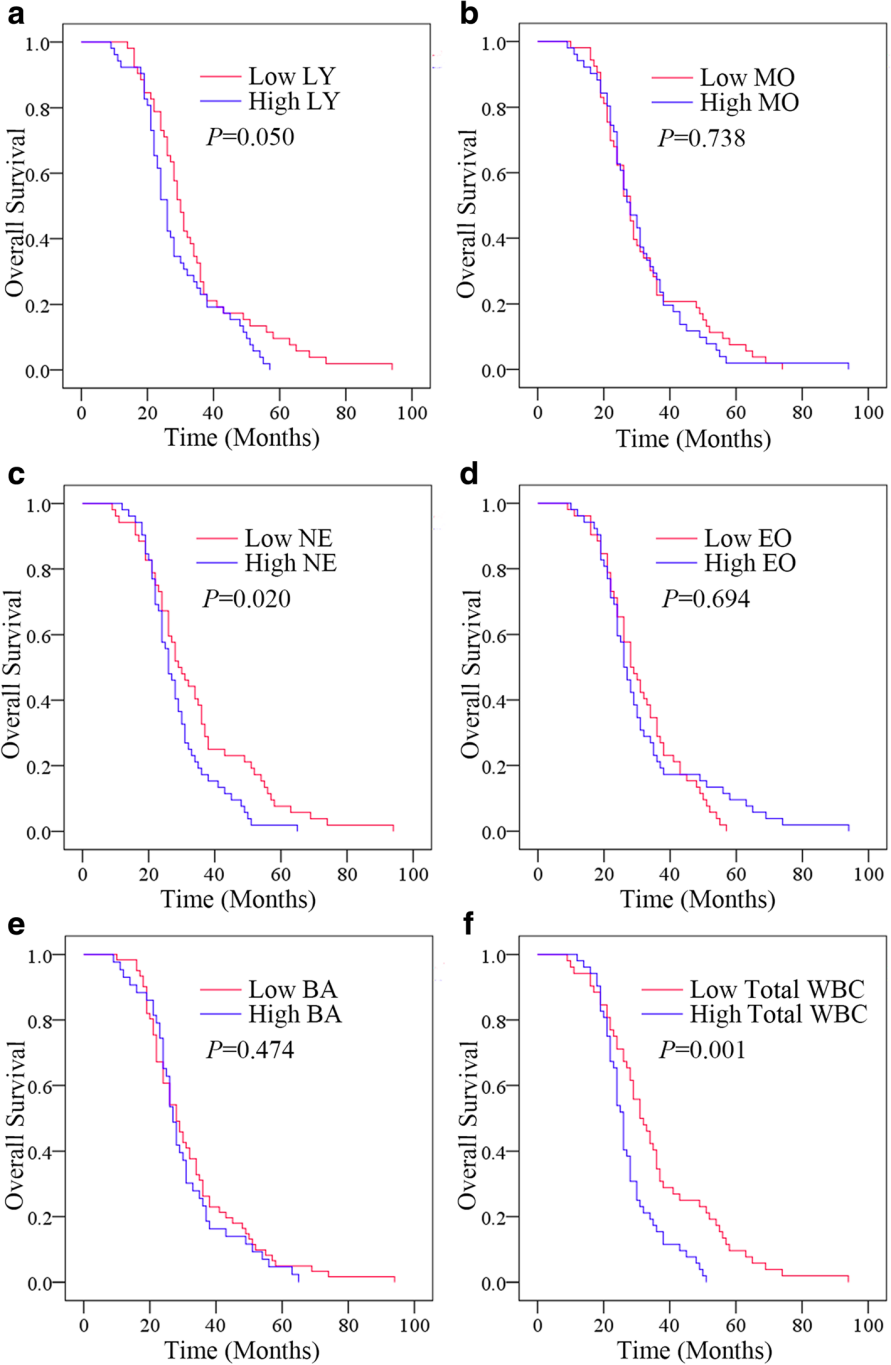
Relationship between status of pre-treatment WBCs and the outcomes. **a** The OS according to pre-treatment LY **b** The OS according to pre-treatment MO. **c** The OS according to pre-treatment NE. **d** The OS according to pre-treatment EO. **e** The OS according to BA. **f** The OS according to pre-treatment total WBC

The Kaplan-Meier plots were used to determine the relationship between OS and the status of pre-treatment LY, MO, NE, EO, BA and total WBC (Fig. 1a-f). The patients were divided into two groups according to the median values of pre-treatment LY (low LY, ≤ 1.535 × 10^9^/L or high LY, > 1.535 × 10^9^/L), pre-treatment MO (low MO, ≤ 0.330 × 10^9^/L or high MO, > 0.330 × 10^9^/L), pre-treatment NE (low NE, ≤ 3.325 × 10^9^/L or high NE, > 3.325 × 10^9^/L), pre-treatment EO (low EO, ≤ 0.090 × 10^9^/L or high EO, > 0.090 × 10^9^/L), pre-treatment BA (low BA, ≤ 0.010 × 10^9^/L or high BA, > 0.010 × 10^9^/L) or pre-treatment total WBC (low WBC, ≤ 5.361 × 10^9^/L or high WBC, > 5.361 × 10^9^/L). The median OS of the high LY group was 26 (95% confidence interval [CI] 23.905–28.095) months, while that of the low LY group was 30 (95% CI 27.352–32.648) months (*P* = 0.050). The median OS of the high NE group was 26 (95% CI 23.352–28.648) months, while that of the low NE group was 29 (95% CI 23.952–34.048) months (*P* = 0.020). The median OS was 26 (95% CI 24.400–27.600) months in the high WBC group and 31 (95% CI 25.952–36.048) months in the low WBC group (*P* = 0.001). The median OS in MO, EO and BA has no significance(all *P >* 0.05) Thus, higher pre-treatment NE and total WBC levels were correlated with poorer prognosis. And pre-treatment levels of LY, MO, EO or BA had no significant effects on OS.

### Effects of surgery on the counts of LY, MO, NE, EO, BA and total WBC

**Fig. 2:**
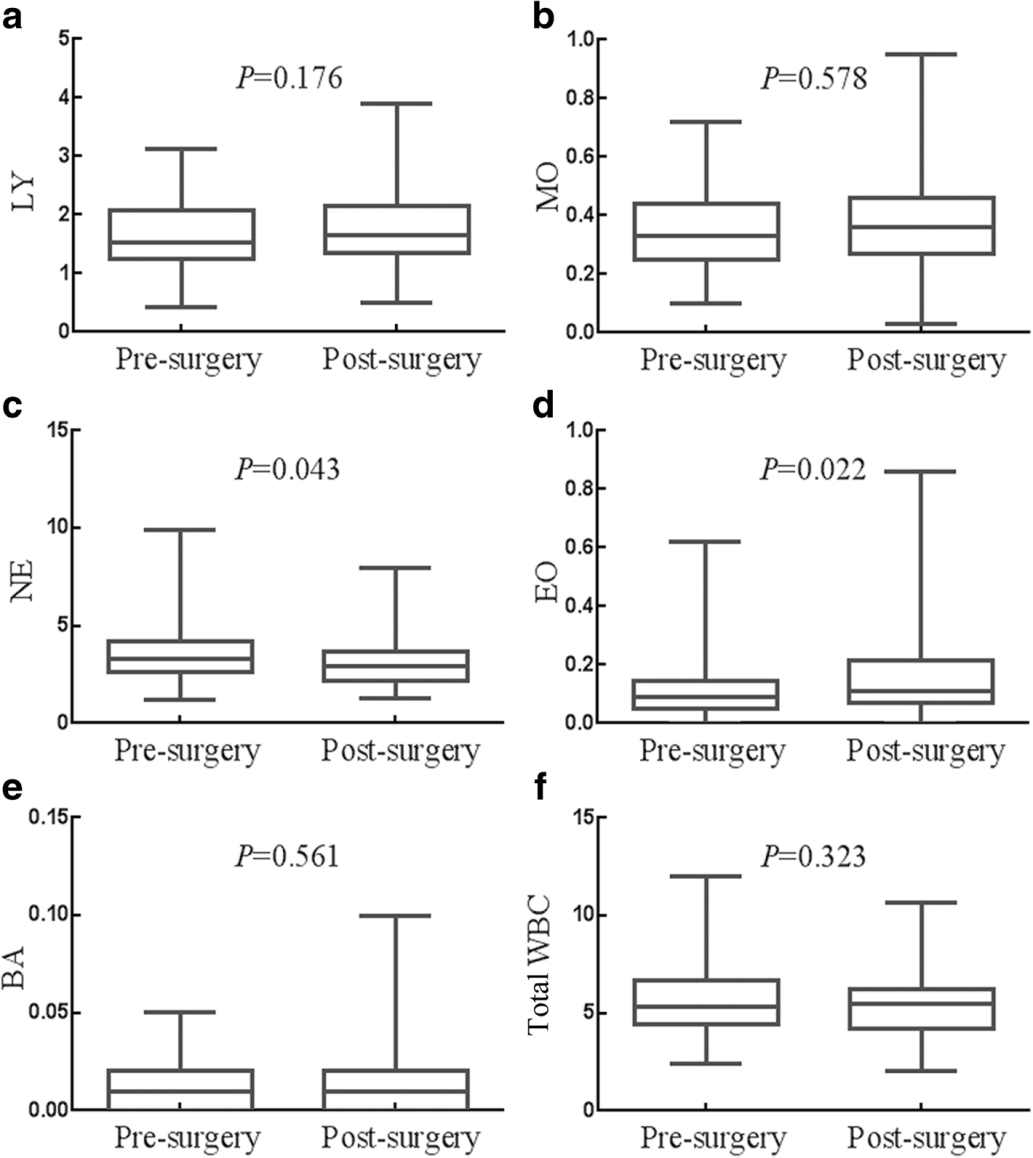
Effects of surgery on the status of WBCs. **a** Surgery had no influence on the count of LY. **b** Surgery had no influence on the count of MO. **c** Surgery decreased the count of NE. **d** Surgery increased the count of EO. **e** Surgery had no influence on the count of BA. **f** Surgery had no influence on the count of total WBC

The effects of surgery on the counts of, LY, MO, NE, EO, BA and total WBC were presented in Fig. 2a-f, respectively. The median count of NE was 3.325 × 10^9^/L (95% CI 2.870 × 10^9^/L-3.535 × 10^9^/L) before surgery, and 2.910 × 10^9^/L (95% CI 2.540 × 10^9^/L-3.145 × 10^9^/L) after surgery (*P* = 0.043). The median count of EO was 0.090 × 10^9^/L (95% CI 0.080 × 10^9^/L-0.100 × 10^9^/L) before surgery, and 0.110 × 10^9^/L (95% CI 0.100 × 10^9^/L-0.140 × 10^9^/L) after surgery (*P* = 0.022). The median count of total WBC and several other WBC like LY, MO and BA has no statistical difference between before surgery and after surgery (all *P* > 0.323). Surgery decreased the count of NE, but increased the count of EO, whereas had no significant impact on the count of LY, MO, BA or total WBC.

### Effects of adjuvant chemotherapyon the counts of LY, MO, NE, EO, BA and total WBC

**Fig. 3:**
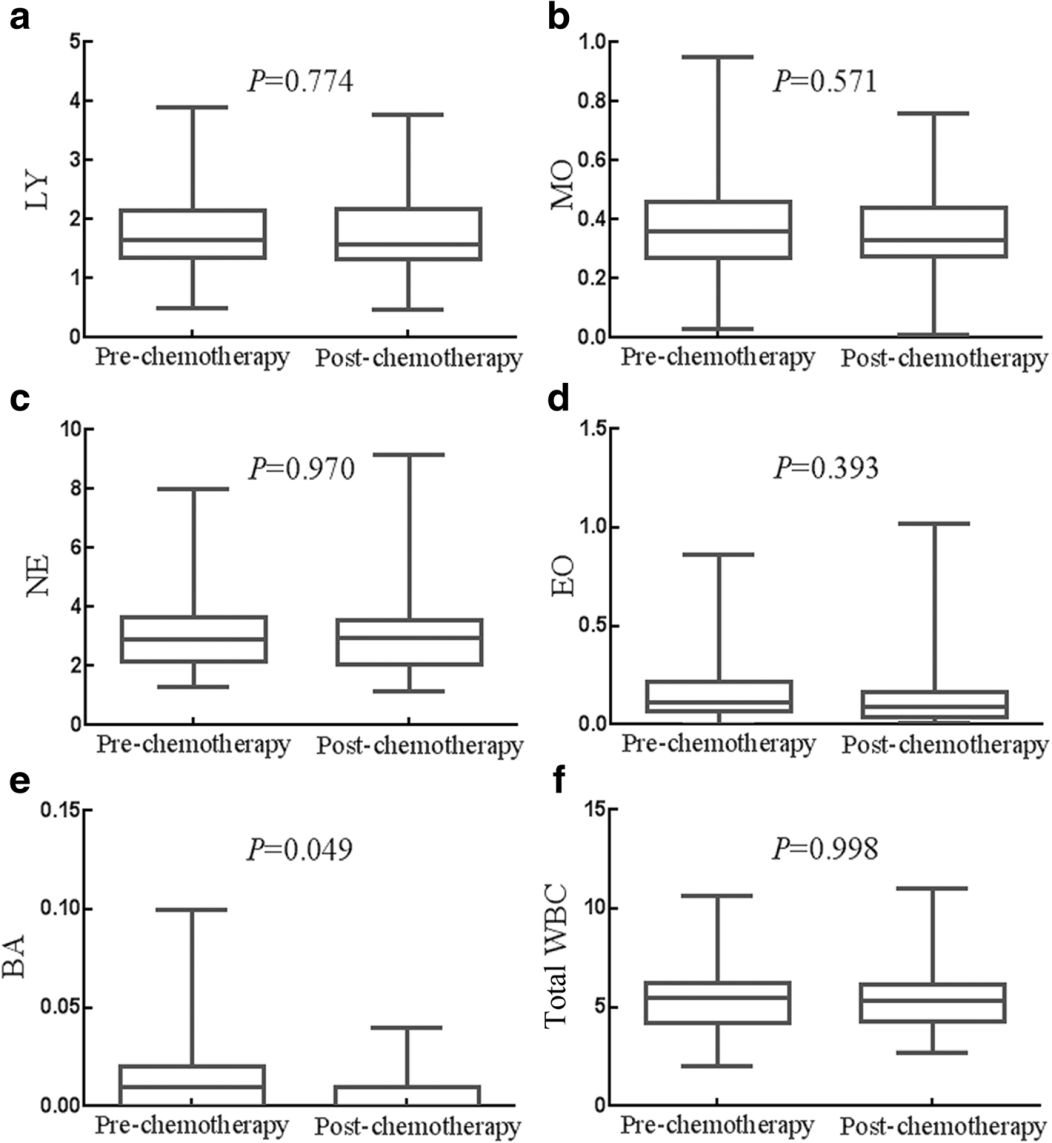
Effects of adjuvant chemotherapy on the status of WBCs. **a** Chemotherapy had no influence on the count of LY. **b** Chemotherapy had no influence on the count of MO. **c** Chemotherapy had no influence on the count of NE. **d** Chemotherapy had no influence on the count of EO. **e** Chemotherapy decreased the count of BA. **f** Chemotherapy had no influence on the count of total WBC

The effects of adjuvant chemotherapy on the counts of LY, MO, NE, EO BA and total WBC were presented in Fig. 3a-f. Only the median count of BA was significant between the before adjuvant chemotherapy group(0.010 × 10^9^/L,95% CI 0.010 × 10^9^/L-0.010 × 10^9^/L) and the after adjuvant chemotherapy group (0.010 × 10^9^/L,95% CI 0.010 × 10^9^/L-0.010 × 10^9^/L) (*P* = 0.049). The median count of total WBC and other WBC such as LY, MO, NE and EO have no significance(all *P* > 0.05). Adjuvant chemotherapy decreased the count of BA, whereas had no significant impact on the count of LY, MO, NE, EO or total WBC.

### Effects of whole course of treatment on the counts of LY, MO, NE, EO, BA and total WBC

**Fig. 4:**
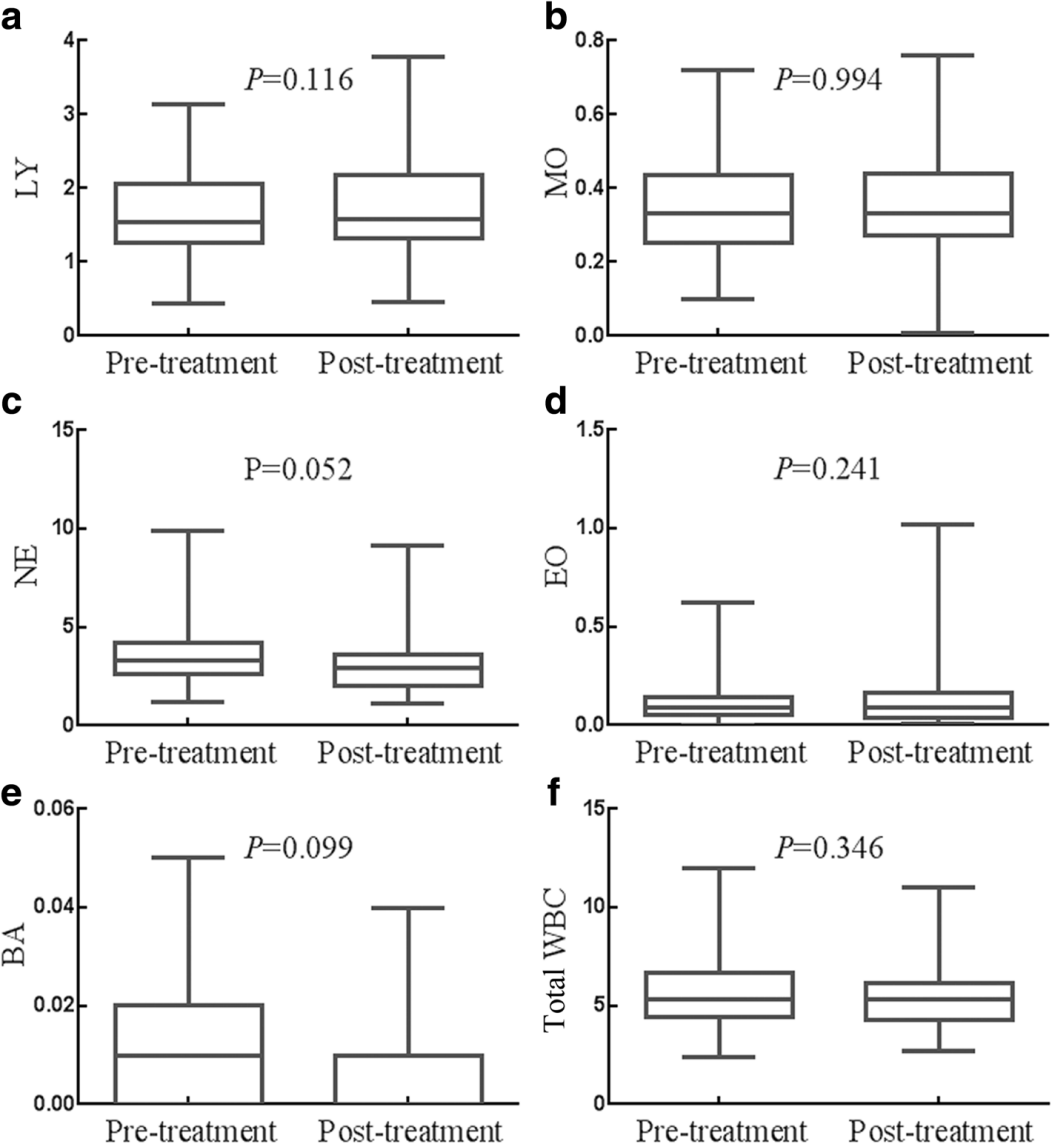
Effects of whole course of treatment on the status of WBCs. **a** Whole course of treatment had no influence on the count of LY. **b** Whole course of treatment had no influence on the count of MO. **c** Whole course of treatment had no influence on the count of NE. (D) Whole course of treatment had no influence on the count of EO. **e** Whole course of treatment had no influence on the count of BA. **f** Whole course of treatment had no influence on the count of total WBC

The impacts of whole course of treatment (surgery and adjuvant chemotherapy) on the count of LY, MO, NE, EO BA and total WBC were presented in Fig. 4a-f. No matter in before treatment group or after treatment group, there is no significant change in the count of LY, MO, NE, BA and total WBC(all *P* > 0.05), which means whole course of treatment (surgery combined with adjuvant chemotherapy) had no significant effect.

### Changes in LY, MO NE, EO, BA and total WBC levels after whole course of treatment could not predict outcomes of resectable GC patients

**Fig. 5:**
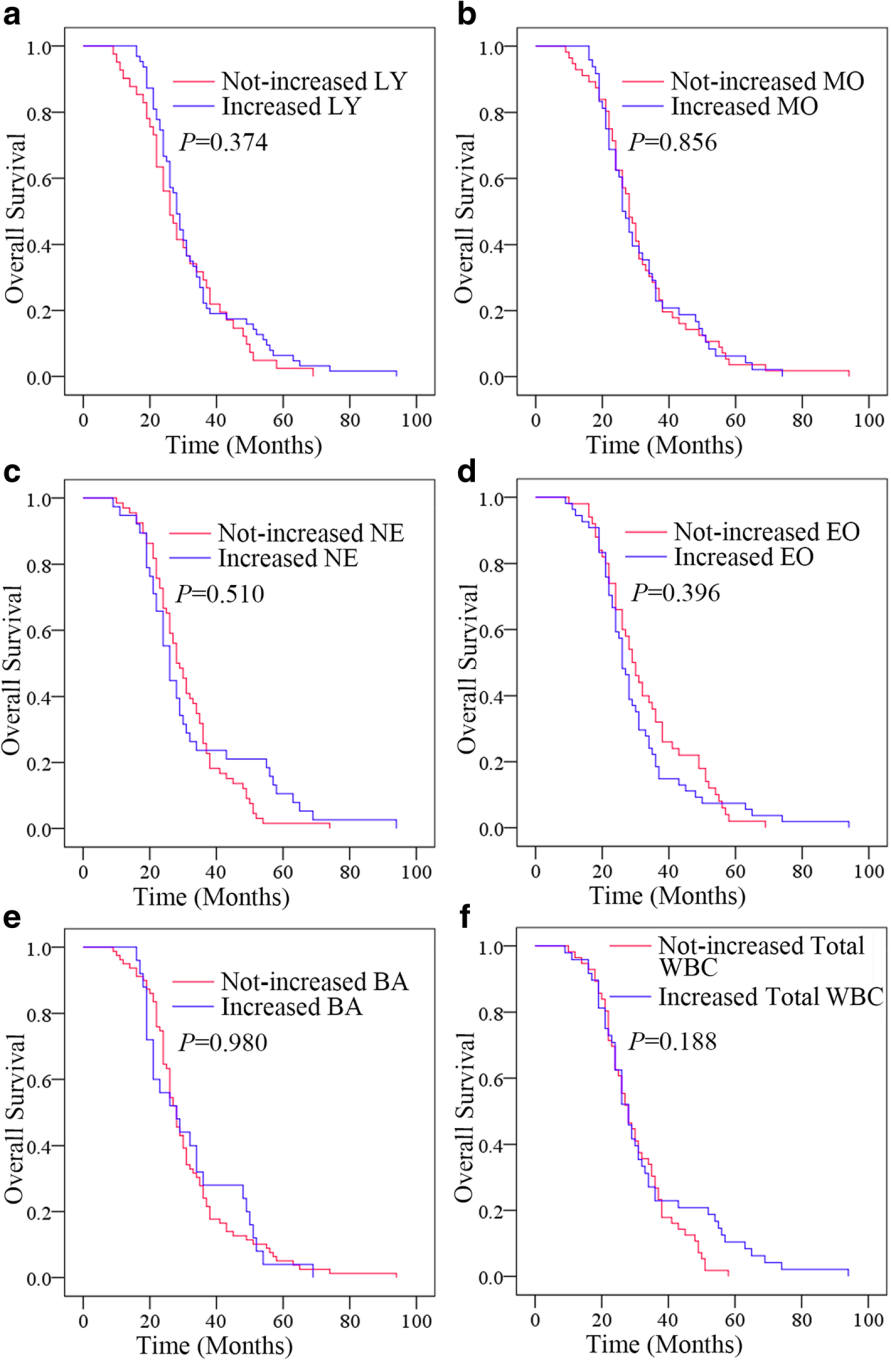
Relationship between changes in status of WBCs after whole course of treatment and the outcomes. **a** The OS according to changes in LY. **b** The OS according to changes in MO. **c** The OS according to changes in NE. **d** The OS according to changes in EO. **e** The OS according to changes in BA. **f** The OS according to changes in total WBC

The Kaplan-Meier plots were used to determine the effect of changes in the blood parameters status for OS (Fig. 5a-f). The median OS of patients whose LY levels increased following whole course of treatment were 28 (95% CI 25.778–30.222) months, while that of the not-increased LY group was 26 (95% CI 21.818–30.182) months (*P* = 0.374). The median OS of patients whose MO and NE levels increased following whole course of treatment were both 26 months, while that of the not-increased MO group and NE group were both 28 months (both *P* > 0.05). The median OS of patients whose EO levels increased following whole course of treatment were 26 (95% CI 23.841–28.159) months, while that of the not-increased EO group was 29 (95% CI 25.040–32.960) months (*P* = 0.396). The median OS of patients whose BA and total WBC levels increased following whole course of treatment were both 28 months, while that of the not-increased BA group and total WBC groupwere both 28 months (both *P* > 0.05). The median OS of patients whose WBC levels increased following whole course of treatment were 28 (95% CI 24.617–31.383) months, while that of the not-increased WBC group was 28 (95% CI 24.343–31.657) months (*P* = 0.188). Thus, changes in LY, MO NE, EO, BA and total WBC levels after therapy had no effects on OS.

### Prognostic factors for resectable GCs

Univariate analyses demonstrated that lymphonodus metastasis (N2) (hazard ratio [HR] 2.116; 95% CI 1.316–3.403; *P* = 0.002), AJCC stage (III) (HR 2.764; 95% CI 1.641–4.657; *P* = 0.000), higher pre-treatment NE level (HR 1.585; 95% CI 1.060–2.371; *P* = 0.025) and higher pre-treatment WBC level (HR 2.023; 95% CI 1.329–3.081; *P* = 0.001) were significant risk factors for a poor prognosis (Table 2). In multivariate analysis, AJCC stag (III) (HR 4.199; 95% CI 1.237–14.247; *P* = 0.021) and higher pre-treatment total WBC count (HR 1.751; 95% CI 1.045–2.935; *P* = 0.033) were found to be independently associated with poor survival.

**Table 2:** Univariate and multivariate logistic regression analysis of risk factors

Risk Factors	Overall Survival (OS)			
	Univariate analysis		Multivariate analysis	
	OR (95% CI)	*P* value	OR (95% CI)	*P* value
Gender				
(Female or Male)	0.714 (0.457–1.115)	0.139	–	–
Age				
(> 60 years or ≤ 60 years)	1.015 (0.992–1.038)	0.192	–	–
Tumor size (cm)				
(> 5 or ≤ 5)	0.848 (0.538–1.337)	0.478	–	–
Depth of invasion	2.062 (0.745–5.705)	0.164	–	–
(T3–4 or T1–2)				
Lymphonodus metastasis				
(N2 or N0–1)	2.116 (1.316–3.403)	0.002**	0.544 (0.166–1.777)	0.313
AJCC stage				
(III orI-II)	2.764 (1.641–4.657)	0.000**	4.199 (1.237–14.24)	0.021*
Pre-treatment LY				
(> 1.535 × 10^9^/L or ≤ 1.535 × 10^9^/L)	1.474 (0.987–2.200)	0.058	–	–
Pre-treatment MO				
(> 0.330 × 10^9^/L or ≤ 0.330 × 10^9^/L)	1.067 (0.722–1.576)	0.745	–	–
Pre-treatment NE				
(> 3.325 × 10^9^/L or ≤ 3.325 × 10^9^/L)	1.585 (1.060–2.371)	0.025*	0.868 (0.522–1.444)	0.586
Pre-treatment EO				
(> 0.090 × 10^9^/L or ≤ 0.090 × 10^9^/L)	0.924 (0.618–1.382)	0.702	–	–
Pre-treatment BA				
(> 0.010 × 10^9^/L or ≤ 0.010 × 10^9^/L)	1.151(0.775–1.709)	0.486	–	–
Pre-treatment total WBC				
(> 5.361 × 10^9^/L or ≤ 5.361 × 10^9^/L)	2.023 (1.329–3.081)	0.001**	1.751 (1.045–2.935)	0.033*
Post/pre-treatment LY ratio				
(> 1 or ≤ 1)	0.839 (0.563–1.249)	0.387	–	–
Post/pre-treatment MO ratio				
(> 1 or ≤ 1)	1.036 (0.703–1.527)	0.860	–	–
Post/pre-treatment NE ratio				
(> 1 or ≤ 1)	0.871 (0.571–1.329)	0.521	–	–
Post/pre-treatment EO ratio				
(> 1 or ≤ 1)	1.180 (0.796–1.748)	0.410	–	–
Post/pre-treatment BA ratio				
(> 1 or ≤ 1)	1.006 (0.638–1.585)	0.980	–	–
Post/pre-treatment total WBC ratio				
(> 1 or ≤ 1)	0.766 (0.508–1.154)	0.202	–	–

*P<0.05; **P<0.01

## Discussion

To date, many studies have reported the relationship between the elevated total WBC count and cancer risks. Thomas P. Erlinger et al. proved that WBC is associated with total cancer mortality after adjustment for age, sex and race [17]. Yong-Jae Lee and colleagues identified that increased WBC count was associated with a higher mortality and incidence risk of colon cancer [18]. Masahiro Lida et al. found that the incidence of GC increased linearly with increasing count of total WBC [19]. Moreover, total WBC count was negatively related to the survival of GC, especially with *H. pylori* (HP) infection [19]. *H. pylori* infection has been shown to increase the production of reactive oxygen metabolites [20, 21], which often cause extensive tissue damage and DNA damage, leading to mutations of oncogenes and tumor suppressors [22, 23]. Therefore, the relationship between total WBC count and survival of GC could be a reflection of the extent of mucosal inflammation induced by *H. pylori* infection [24]. In our present study, we found that higher pre-treatment total WBC status was associated with worse OS, in consisting with previous studies. Univariate analysis demonstrated that higher pre-treatment total WBC count was a significant risk factors and multivariate analysis demonstrated that total WBC count was an independent prognostic marker for a poor prognosis. However, neither surgery nor adjuvant chemotherapy could affect total WBC count. Post/pre-treatment ratio of total WBC had no significant effects on OS either.

Previous studies have confirmed that peripheral blood LY count is an independent prognostic factor for multiple cancers, such as breast [25], pancreatic [26], lung [27], cervical [28] and gallbladder cancers [29]. In cell-mediated anti-tumor immune response, LY has a crucial role in many cancers [30, 31]. Tumor-infiltrating lymphocytes (TILs) have a positive effect on OS in colorectal and ovarian cancers [32, 33], which may due to the apoptosis induced by tumor cells and the activation of specific CD8+ T cells. In addition, CD4+ T lymphocytes play a key role by secreting cytokines such as IL-2, which is required for CD8+ T lymphocytes proliferation and growth. Previous studies indicated that, activation of CD4+ T cells are required for immunization of CD8+ T cells against cancer [34]. However, in the present study, we found that LY count was not associated with OS in resectable GC patients.

Inflammation-induced carcinogenesis is caused by several processes, including genotoxicity, aberrant tissue repair, proliferation, invasion and metastasis [35]. MO, as a marker of systemic inflammatory response, may thus be a predictive factor for various inflammation-related cancers. In many previous studies, MO has been proved to be negatively related to OS of tumor patients, such as pancreatic cancer and hepatocellular carcinoma [36, 37]. Multiple mechanisms might be involved in the relationship between MO and tumor prognosis. As mentioned above, the inflammatory response has laid an important foundation in the development of cancer. MO, as an important component of the adaptive and innate immune system, can both facilitate angiogenesis and tumorigenesis. MO can also secret soluble mediators and promote the growth of cancer [38]. In addition, Evani et al. found that MO can promote proliferation of breast cancer by improving adhesion of tumor cells [39]. Besides, MO can differentiate into tumor-associated macrophage (TAM), which plays a major part in tumor microenvironment through promoting tumor progression, metastasis, angiogenesis, migration and immune escape [40]. However, in our present study, neither surgery nor adjuvant chemotherapy had significant effects on MO count. In addition, pre-treatment or post/pre-treatment ratio of MO had no effects on OS, either.

Chronic inflammation is essential for cancer growth and metastasis. NE is one of the well-known marker of systemic inflammatory response. Increasing evidences show that it is also a predictor of poor prognosis in a variety of malignant tumors [10, 11, 41, 42]. It has been reported that activation of endogenous or exogenous pathways could mobilize transcription factors and inflammatory mediators, which may led to recruitment of inflammatory cells, including NE [43]. In our present study, we found that NE count could be down-regulated by surgery. In addition, pre-treatment NE count was negatively related to OS, and univariate analysis indicated that higher pre-treatment NE count was a significant risk factors for a poor prognosis. Basing on changes in individual NE count levels, post/pre-treatment ratio of NE had no significant effects on OS.

Higher EO count has been observed in several malignant tumors, such as colorectal [44], breast [45], cervical [46], oral squamous [47] and prostate cancers [48]. The relationship between EO and prognosis is still indefinite. In colorectal and prostate cancers, EO infiltration has been linked to a favourable prognosis [44, 48]. However, EO appears to be an independent and significant unfavorable prognostic factor in adult T cell leukaemia/lymphoma, chronic eosinophilic leukemia and Hodgkin’s lymphoma [49–51]. The growth-promoting effect EO could be executed through secreting several pro-angiogenic factors, including VEGF, fibroblast growth factor-2, and IL-8 [52]. Our present study revealed that surgery increased the level of EO count. However, neither pre-treatment level nor post/pre-treatment ratio of EO count was related to the prognosis of resectable GC patients.

Researches on the functions of BA were hindered by their rarity, difficulty in identifying, and lack of useful analytical tools [14]. Existing researches have focused on the role of BA playing in parasite allergy, immediate hypersensitivity and autoimmunity [16]. Moreover, as a type of immune cells, BA is demonstrated to be negatively associated with outcomes of pancreatic cancer [53]. Additionally, in a mouse model of metastatic breast cancer by implanting 4 T1 cells into the mammary fat pads, some reseacheres identified that the presence of BA predicted tumor growth. In the present study, chemotherapy down-regulated BA status. While, pre-treatment level and post/pre-therapeutic ratio of BA had no significant effects on OS.Taken together, our present investigation showed that higher pre-treatment level of NE and total WBC counts were correlated with worse prognosis in resectable GC. These noninvasive, simple and low-cost biomarkers may be prognostic indicators. The limitations of our study may include its retrospective design, recruitment of patients from single center, and insufficiency of case number. To eliminate the differences in the general performance of patients, we studied each case thoroughly and excluded patients with chronic diseases such as inflammatory bowel diseases and rheumatic diseases, etc.al. Eventually, only a limited number of 104 cases were obtained. Likely, our results were consistent with the previous studies, and we believed our results were highly reliable. Of course, we would conduct a multi-center study and collect more cases data to further explore the conclusion in the future.

## Conclusions

Our study demonstrated that pre-treatment NE and total WBC counts were negatively correlated with overall survival (OS). Surgery significantly decreased the level of NE count, and adjuvant chemotherapy significantly decreased the level of BA. Univariate analysis indicated that AJCC stage (III) and higher level of pre-treatment total WBC count were prognostic factors affecting OS. Multivariate Cox regression analysis revealed that AJCC stage (III) and higher level of pre-treatment total WBC count were independent prognostic factors. Therefore, we suggested that pre-treatment NE count and pre-treatment total WBC count may be potential prognostic factors for the prognostic evaluation of GCs.

## Abbreviations

AJCC: The American Joint Committee on Cancer
BA: Basophil
CT: Computed tomography
EDTA: Ethylenediaminetetraacetic acid
EO: Eosinophil
GCs: Gastric cancers
HP: *H. pylori*
LY: Lymphocyte
MO: Monocyte
NE: Neutrophil
OS: Overall survival
RECIST: Response Evaluation Criteria in Solid Tumors
TAM: Tumor-associated macrophage
TILs: Tumor-infiltrating lymphocytes
TNM: Tumor-nodulus-metastases
WBC: White blood cell
